# Long-term prehypertension treatment with losartan effectively prevents brain damage and stroke in stroke-prone spontaneously hypertensive rats

**DOI:** 10.3892/ijmm.2013.1583

**Published:** 2013-12-10

**Authors:** DE-HUA HE, LIANG-MIN ZHANG, LI-MING LIN, RUO-BING NING, HUA-JUN WANG, CHANG-SHENG XU, JIN-XIU LIN

**Affiliations:** 1 Department of Cardiology, The First Affiliated Hospital of Xiamen University, Fuzhou, Fujian, P.R. China; 2 Department of Cardiology, The First Clinical Medical College of Fujian Medical University, Fuzhou, Fujian, P.R. China; 3 Department of Cardiology, Affiliated Hospital of Putian College, Fuzhou, Fujian, P.R. China; 4 Fujian Institute of Hypertension, Fuzhou, Fujian, P.R. China

**Keywords:** prehypertension, losartan, amlodipine, stroke-prone spontaneously hypertensive rats

## Abstract

Prehypertension has been associated with adverse cerebrovascular events and brain damage. The aims of this study were to investigate i) whether short- and long-term treatments with losartan or amlodipine for prehypertension were able to prevent blood pressure (BP)-linked brain damage, and ii) whether there is a difference in the effectiveness of treatment with losartan and amlodipine in protecting BP-linked brain damage. In the present study, prehypertensive treatment with losartan and amlodipine (6 and 16 weeks treatment with each drug) was performed on 4-week-old stroke-prone spontaneously hypertensive rats (SHRSP). The results showed that long-term (16 weeks) treatment with losartan is the most effective in lowering systolic blood pressure in the long term (up to 40 weeks follow-up). Additionally, compared with the amlodipine treatment groups, the short- and long-term losartan treatments protected SHRSP from stroke and improved their brains structurally and functionally more effectively, with the long-term treatment having more benefits. Mechanistically, the short- and long-term treatments with losartan reduced the activity of the local renin-angiotensin-aldosterone system (RAAS) in a time-dependent manner and more effectively than their respective counterpart amlodipine treatment group mainly by decreasing AT1R levels and increasing AT2R levels in the cerebral cortex. By contrast, the amlodipine treatment groups inhibited brain cell apoptosis more effectively as compared with the losartan treatment groups mainly through the suppression of local oxidative stress. Taken together, the results suggest that long-term losartan treatment for prehypertension effectively protects SHRSP from stroke-induced brain damage, and this protection is associated with reduced local RAAS activity than with brain cell apoptosis. Thus, the AT1R receptor blocker losartan is a good candidate drug that may be used in the clinic for long-term treatment on prehypertensive populations in order to prevent BP-linked brain damage.

## Introduction

The term ‘prehypertension’ was first introduced in JNC-7 in 2003 to define individuals whose systolic blood pressure (BP) levels are between 120 and 139 mmHg or diastolic BP between 80 and 89 mmHg (1,2). The aim of this definition by the Joint National Committee on Prevention, Detection, Evaluation, and Treatment of High Blood Pressure is to target the population with hypertension who has a higher-than-normal BP and is at a higher-than-normal risk of developing cardiovascular disease. Results of previous studies have shown that individuals with prehypertension had an increased risk of developing hypertension compared with the normotensive population (3,4), and had an increased age-related risk of cardiovascular and cerebrovascular diseases including stroke and carotid atherosclerotic plaque (5,6). As with hypertension, one of the target organs for prehypertension is brain. Findings of the Framingham Heart Study suggested that prehypertension caused impairment to the structural integrity of white matter and shrinkage of gray matter in young populations even before these individuals developed hypertension, and the damage was associated with premature aging but not stroke (7). Thus, it appears that BP-associated brain damage may start at a relatively younger age than expected and may continue over a long period of time if the abnormal BP is not controlled or corrected in time.

There has been controversy regarding whether antihypertensive drug treatment should be applied to prehypertensive populations. Findings of the TROPHY study showed that two years of treatment with candesartan, an angiotensin receptor blocker, had a short-term effect on preventing the progression of prehypertension to hypertension compared with the placebo group (8). However, the long-term benefit following drug withdrawal remains to be clarified (8). Thus, although it is important to lower BP during the prehypertension period to prevent or decrease the occurrence of cardiovascular diseases at mid- or late-life, how to efficiently achieve this long-term objective remains to be determined. Lifelong drug treatment for prehypertension is required, as in the case for hypertension, however, a number of factors should be considered for any given prehypertensive individual, such as the tolerance of drug, concomitant diseases and drug treatment. Adoption of a healthier lifestyle such as an increase in physical activity and diet modification has been proven effective, however, it is difficult to be realized, particularly in the young and middle-aged prehypertensive populations (9). The aforementioned potential organ damages caused by prehypertension have therefore led to a search for an appropriate drug for the long-term treatment of prehypertension.

In the clinic, angiotensin II (Ang II) receptor antagonists and calcium channel blockers are two classes of drugs that have been widely used to treat patients with hypertension. In spontaneously hypertensive rats (SHR), prehypertensive treatment with losartan maintained cardiac protection until the age of 48 weeks, but not with hydralazine, a general vasodilator (10). In a previous study it was suggested that transient losartan treatment on SHR produced improved cardioprotective and renoprotective effects compared with amlodipine in the long-term (11). The abovementioned studies demonstrated that different drugs have different impacts on BP-associated organ damage. By contrast, long-term treatment with losartan on salt-loaded stroke-prone SHR (SHRSP) reduced cerebrovascular damage and provided full protection against mortality (12). However, whether transient administration of any of these drugs leads to long-term protection against brain damage associated with prehypertension, and whether there is any difference in the effectiveness of these drugs in prehypertensive treatment against cerebrovascular impairment remain to be determined.

In the present study, prehypertensive administration of losartan and amlodipine was performed for 6 and 16 weeks, respectively, on SHRSP. Continuous brain damage up to 40 weeks among these groups was monitored by activity observations, histological and biochemical examinations. Losartan is therefore a preferred alternative to amlodipine for prehypertensive treatment in lowering long-term BP as well as effectively limiting brain damage associated with prehypertension in SHRSP. Therefore, losartan is a good candidate drug that may be used in the clinic to treat prehypertensive patients who have an increased risk of developing adverse cerebrovascular events.

## Materials and methods

### Animals

SHRSP and Wistar Kyoto rats (WKY) were purchased from SLAK Laboratory Animal. A total of 120 male, 4-week-old SHRSP were randomly divided into 5 groups (n=24 rats per group): i) SHRSP-Veh: SHRSP treated with saline, 2 ml/kg; ii) SHRSP-Los6: SHRSP treated with 20 mg/kg/day losartan for 6 weeks; iii) SHRSP-Los16: SHRSP treated with 20 mg/kg/day losartan for 16 weeks; iv) SHRSP-Aml6: SHRSP treated with 10 mg/kg/day amlodipine for 6 weeks; v) SHRSP-Aml16: SHRSP treated with 10 mg/kg/day amlodipine for 16 weeks. Twenty-four untreated WKY rats were used as the control group. The rats were followed up until they were 40 weeks of age. Systolic blood pressure (SBP) was measured using the tail-cuff method at different ages (weeks). The clinical scores of stroke of each group were evaluated at ages of 10, 20 and 40 weeks, based on the symptomatological classification (13) with slight modifications as follows: level 0, normal activity; level 1, slightly decreased activity and/or slightly agitated; level 2, significantly decreased activity and/or highly agitated; level 3, lethargic and depression-like symptoms; level 4, paralyzed (either one or two sides). Rats (4 animals per cage) were housed in a room with controlled temperature (23±2°C) under a 12-h light/dark cycle. The animals had access to standard food and tap water *ad libitum*. Procedures were approved by the Animal Ethics Committee of Fujian Medical University and performed in accordance with institutional guidelines.

### Reagents

The reagents used in the present study were: Losartan (Hanzhou Merck Sharp & Dohme), amlodipine (Pfizer), antibodies against AT1R, AT2R, gp91^phox^ and SOD1 (Abcam), anti-β-actin antibody and HRP-conjugated secondary antibody (ZSGB-Bio). ^125^I-labeled angiotensin II and aldosterone (Ald) radioimmunoassay kits (Beijin North Institute of Biological Technology), and in Situ Cell Death Detection Kit POD (Roche) were also utilized.

### Radioimmunoassay

Ang II and Ald levels in rat brain were measured using radioimmunoassay kits according to the manufacturer’s instructions.

### Histology, apoptosis staining and transmission electron microscopy (TEM)

For regular histology, rat brains were fixed in 10% formaldehyde and sectioned at 10 μm. The sections were stained with hematoxylin and eosin (H&E) according to the standard protocol. TUNEL staining was performed according to the manufacturer’s instructions. TEM was performed using standard procedures.

### Western blot analysis

Western blots were performed as previously described (11). Briefly, 100 μg of protein lysates purified from brains of rats from each group was loaded into SDS denatured gel, transferred to PVDF membrane, and probed with designed antibodies. Protein bands were visualized by electrochemiluminescence on high-performance chemiluminescence film and quantified using image analysis software. The results were expressed as a ratio of gp91^phox^, SOD, AT1R, and AT2R over β-actin.

### Statistical analysis

ANOVA and the unpaired Student’s t-test were applied to determine statistical significance between groups when applicable and shown in each figure legend. To evaluate the significance of clinical scores of stroke, the Kruskal-Wallis H test was used to analyze the significance of data distribution in the six groups, followed by the Mann-Whitney U test for comparison between two groups. Levels of statistical significance were defined as P<0.05.

## Results

### Changes in BP in rats at different time points

**Figure 1:**
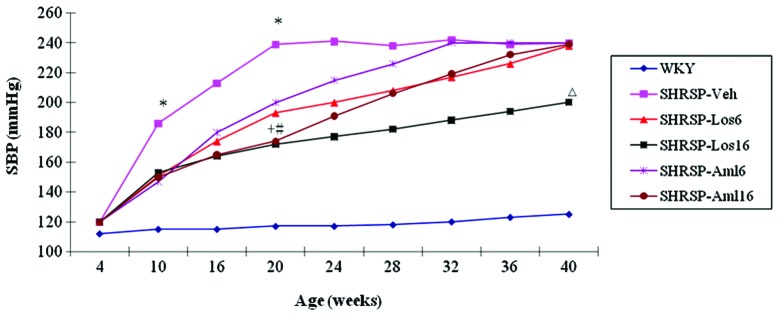
Changes in systolic blood pressure (SBP) of Wistar Kyoto rats (WKY), stroke-prone spontaneously hypertensive rats (SHRSP)-Veh and the four SHRSP treatment groups at different ages (weeks). ^*^P<0.05, compared with SHRSP drug treatment groups; ^+^P<0.05, compared with SHRSP-Los6; ^#^P<0.05, compared with SHRSP-Aml6; ^△^P<0.05, compared with the three remaining drug treatment groups and SHRSP-Veh.

To determine whether and how losartan and amlodipine would affect prehypertension-associated brain damage, we employed SHRSP as a model system. Animals were divided into six groups (n=24 animals per group): WKY, vehicle (SHRSP-Veh), SHRSP-Los6 (6 weeks of losartan treatment), SHRSP-Los16 (16 weeks of losartan treatment), SHRSP-Aml6 (6 weeks of amlodipine treatment), and SHRSP-Aml16 (16 weeks of amlodipine treatment). Animals were treated at 4 weeks of age, when the BP of SHRSP was higher than normal, but lower than the defined high BP, which resembles that of prehypertension in humans (11). We first measured the SBP of these animals from each group at different ages (weeks) up to 40 weeks (Fig. 1). At 4 weeks age, the starting BP of SHRSP of each group was higher than that of the control (WKY) group, but lower than that of the high BP group after maturation, suggesting that SHRSP at the age of 4 weeks serves as a good model system for investigation of prehypertension. At 10 and 20 weeks, a significant decrease in SBP in all the treatment groups was observed compared with SHRSP-Veh (P<0.05). However, at 20 weeks, the SBP of animals in the SHRSP-Los16 or SHRSP-Aml16 groups was significantly lower than those of animals in the SHRSP-Los6 and SHRSP-Aml6 (P<0.05, SHRSP-Los16 vs. SHRSP-Los6, and P<0.05, SHRSP-Aml16 vs. SHRSP-Aml6) groups, suggesting that long-term treatment of either of the two drugs exerted more effects compared with a short-term treatment. At 40 weeks, only SHRSP-Los16 animals maintained markedly lower SBP compared with the remaining four SHRSP groups (P<0.05), while the animals of the three drug treatment groups, SHRSP-Los6, SHRSP-Aml6, and SHRSP-Aml16, showed no difference in SBP compared with SHRSP-Veh.

The results therefore showed that i) 6 weeks (short-term) treatment of losartan or amlodipine in SHRSP was able to decrease BP and maintain it at a low level for a short period of time (until 20 weeks, i.e., 10 weeks after administration withdrawal), ii) 16 weeks (long-term) treatment of losartan or amlodipine in SHRSP caused a more significant decrease in BP at 20 weeks than the 6-week treatment groups, and iii) SHRSP-Los16 maintained lower BP more effectively than SHRSP-Aml16 for a longer period of time (up to 40 weeks).

### Clinical scores of stroke of the six groups

SHRSP is a proven model that may develop stroke after maturation. To determine whether prehypertension treatment with losartan or amlodipine had any impact on the occurrence of stroke, we evaluated the clinical scores of stroke from each group at the age of 10, 20 and 40 weeks based on the symptomatological classification (13) with slight modification. At 10 and 20 weeks, no behavioral abnormalities were observed in the animals of any of these groups (data not shown). At age of 40 weeks, the number of animals that had died in the SHRSP-Veh, SHRSP-Aml6 and SHRSP-Aml16 groups was 3, 2 and 1, respectively. The clinical score of each group was evaluated and presented in Table I. The SHRSP-Los6 and SHRSP-Los16 groups showed a significant improvement in the daily activity of animals compared with that of the SHRSP-Veh group, although they were worse compared with the WKY group. Animals in the SHRSP-Aml6 and SHRSP-Aml16 groups did not show any significant difference in this evaluation compared with the SHRSP-Veh group. Thus, prehypertension treatment with losartan is more effective in decreasing stroke occurrence and in improving quality of life than amlodipine, in the animal model.

### Changes in the brain structure of the animals in the six groups

**Figure 2:**
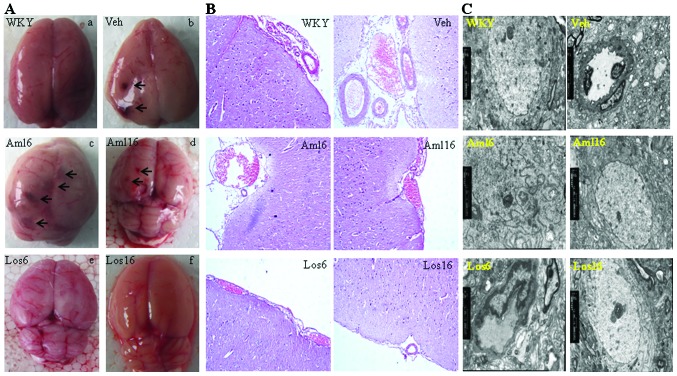
Changes in the brain structure of Wistar Kyoto rats (WKY), stroke-prone spontaneously hypertensive rats (SHRSP)-Veh and the four drug treatment SHRSP groups at the age of 40 weeks. (A) A global view of brains dissected from rats of each group investigated. Arrows point to the bleeding spots. Note that no obvious bleeding spots were observed in the brains from the SHRSP-Los6 and -Los16 groups. (B) Hematoxylin and eosin (H&E) staining shows bleeding and/or disorganized brain cells in the SHRSP-Veh, SHRSP-Los6, and two amlodipine treatment groups, but not the SHRSP-Los16 group. (C) Ultrastructural analysis of brain tissues from the six groups by transmission electron microscopy shows swollen mitochondria and disrupted cristae in SHRSP-Veh. Similar pathological changes are present in the SHRSP-Aml6, SHRSP-Aml16 and SHRSP-Los6 groups, but not in the SHRSP-Los16 and WKY groups. (A–C) Representative data are shown.

To gain a better understanding of the structural basis underlying brain damage in SHRSP and improvement by treatments, we first globally viewed the brains from each group at the age of 40 weeks (Fig. 2A): WKY rat showed normal brain structure without edema and bleeding, and the midline was straight and not shifted. SHRSP-Veh rat brain exhibited shifted midline, edema and significant bleeding spots (Fig. 2A–b, arrows). SHRSP-Aml6 and -Aml16 brains showed pathological changes similar to those observed in SHRSP-Veh group (Fig. 2A–c and -d). However, SHRSP-Los6 and SHRSP-Los16 showed improved brain morphology similar to that of the WKY rats (Fig. 2A–e and -f). Thus, either short-term (6 weeks) or long-term (16 weeks) treatment with losartan protects SHRSP brain from prehypertension-associated damage.

Histological analysis by H&E staining on the sections of brains from these groups further corroborated the above findings. SHRSP-Los16 showed relatively normal structure in brain and showed no bleeding, similar to the WKY rats, while the rats from all the remaining groups had internal bleeding in brains (Fig. 2B). The ultrastructural changes on the sections of brains prepared from SHRSP vehicle and treatment groups were examined and compared with those of the WKY rats at the age of 40 weeks. WKY rat brain showed relatively normal structure of mitochondria and closely connected brain cells, while the examination of the brain section of SHRSP-Veh showed swollen mitochondria that contained vacuoles with disintegrated cristae, and a loose connection between brain cells (Fig. 2C). Similar to the findings in Fig. 2A and B, the SHRSP-Los16 group showed a much better improvement in the brain ultrastructure than the SHRSP-Los6 group, while the amlodipine treatment groups showed comparable pathological changes similar to those of the SHRSP-Veh group (Fig. 2C). These data collectively suggest that SHRSP-Los16 has the best protective effects among the treatment groups in terms of maintaining normal brain structure.

### Apoptosis in the brain of the five treatment groups and the control group

**Figure 3:**
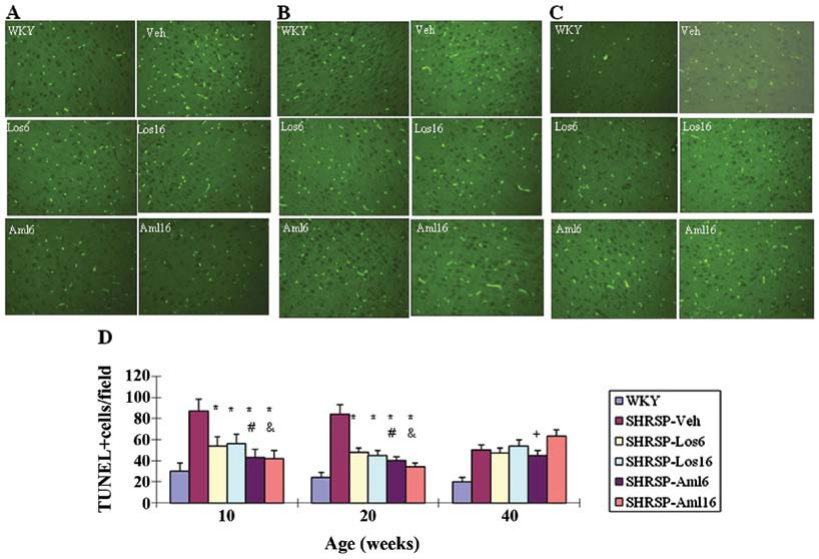
Comparison of apoptosis in the brains of Wistar Kyoto rats (WKY), stroke-prone spontaneously hypertensive rats (SHRSP)-Veh and the four drug treatment groups at different ages (weeks). TUNEL staining was performed on 5 μm sections of brains prepared from these experimental groups as indicated. TUNEL-positive cells were scored from three independent fields per sample, n=8/group, as shown at (A) 10 weeks, (B) 20 weeks, (C) 40 weeks; (D) statistical analysis of A–C. ^*^P<0.05 compared with SHRSP-Veh; ^#^P<0.05, compared with SHRSP-Los6; ^&^P<0.05, compared with SHRSP-Los16. Comparisons were made between groups of the same age as indicated. Note that there is a significant difference between WKY and any of the SHRSP groups.

To determine the molecular basis underlying pathological changes observed in SHRSP-Veh and other treatment groups, we investigated apoptosis on brain sections prepared from WKY, SHRSP-Veh and the four drug treatment groups at different ages (10, 20 and 40 weeks). The number of apoptotic cells was significantly reduced in each of the four treatment groups at 10 and 20 weeks compared with that in the SHRSP-Veh group (P<0.05) (Fig. 3). At 10 and 20 weeks, SHRSP-Aml6 and SHRSP-Aml16 were more effective in inhibiting apoptosis compared with their respective counterparts, respectively (P<0.05, SHRSP-Aml6 vs. SHRSP-Los6; P<0.05, SHRSP-Aml16 vs. SHRSP-Los16). However, at 40 weeks, all the treatment groups and SHRSP-Veh showed a comparable number of apoptotic cells.

Apoptosis was at least partly attributable to increased oxidative stress (14), and increased oxidative stress and free radicals were observed in the brains of SHRSP (15,16).

### Changes in the levels of gp91phox and superoxide dismutase (SOD) in the cerebral cortex

**Figure 4:**
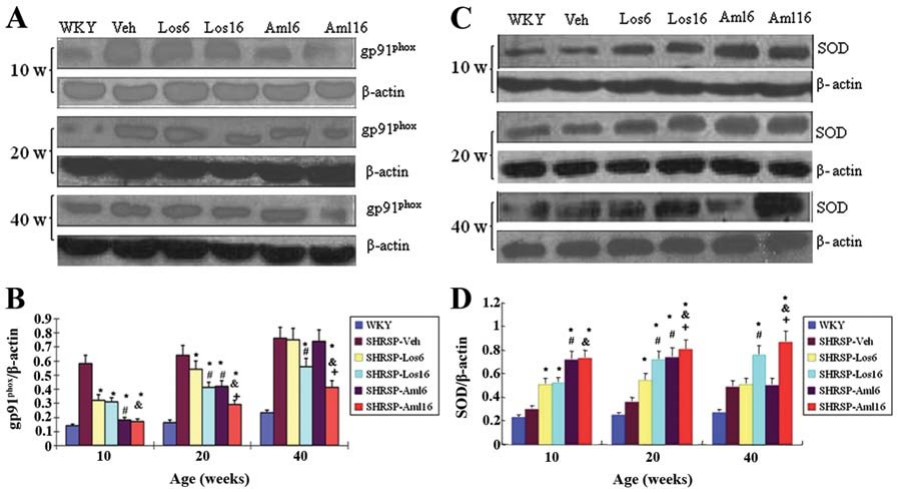
Changes in the levels of gp91^phox^ and superoxide dismutase (SOD) in the cerebral cortex of Wistar Kyoto rats (WKY), stroke-prone spontaneously hypertensive rats (SHRSP)-Veh and the four drug treatment groups at different ages (weeks). (A and B) Western blot was performed on protein lysates extracted from brains of rats from each group at different ages as indicated (A), and the statistical analysis is shown in (B). β-actin served as the control, n=8/group. (C and D) Western blot was performed as in (A), but the blot was reacted with anti-SOD antibody (C), and the statistical analysis is shown in (D), n=5/group. ^*^P<0.05, compared with SHRSP-Veh; ^#^P<0.05, compared with SHRSP-Los6; ^&^P<0.05, compared with SHRSP-Los16; ^+^P<0.05, SHRSP-Aml6. The comparisons were made between groups of the same age as indicated. Note that there is a significant difference between WKY and any of the SHRSP groups.

To explore whether the treatment groups with prehypertension had any impact on oxidative stress, we performed western blots in brain of all the groups to evaluate the levels of gp91^phox^, a heme binding subunit of the superoxide-generating NADPH oxidase (17), and an important factor in promoting oxidative stress-associated brain damage (18), and superoxide dismutase (SOD), an anti-oxidative factor (19). At 10 weeks, the treatment groups showed a significant decrease in gp91^phox^ (Fig. 4A and B, P<0.05), while the two amlodipine treatment groups were more effective than their respective counterparts (P<0.05, SHRSP-Aml6 vs. SHRSP-Los6, and P<0.05, SHRSP-Aml16 vs. SHRSP-Los16). At 20 weeks, while all the treatment groups remained effective in lowering gp91^phox^ levels in the brains, two long-term treatment groups were more effective compared with their respective short-term treatments. Among these groups, SHRSP-Aml16 was the most effective at this time (P<0.05, SHRSP-Aml16 vs. SHRSP-Los16). At 40 weeks, the levels of gp91^phox^ in the brains of the two short-term treatment groups returned to the comparable levels similar to that of SHRSP-Veh, while the pattern of the decreased levels of gp91^phox^ in the two long-term treatment groups was similar to the one shown at 20 weeks.

For SOD levels, the treatment groups showed a significant increase compared with that of the SHRSP-Veh group at 10 and 20 weeks (Fig. 4C and D, P<0.05), while SOD levels were higher in the two amlodipine treatment groups compared with their respective counterparts (P<0.05, SHRSP-Aml6 vs. SHRSP-Los6, and P<0.05, SHRSP-Aml16 vs. SHRSP-Los16). At 20 weeks, the long-term treatment groups had a greater impact than their respective short-term treatment groups (P<0.05, SHRSP-Los16 vs. SHRSP-Los6; P<0.05, SHRSP-Aml16 vs. SHRSP-Aml6). It is noteworthy that the SOD levels in SHRSP-Aml16 were significantly higher than those in SHRSP-Los16 (P<0.05). At 40 weeks, the SOD levels in two short-term treatment groups returned to levels similar to those of SHRSP-Veh, however, the two long-term treatment groups maintained higher SOD levels than those of SHRSP-Veh. Similar to 20 weeks, the SOD levels in SHRSP-Aml16 remained significantly higher than those in SHRSP-Los16 (P<0.05, SHRSP-Aml16 vs. SHRSP-Aml6). Collectively, these findings suggested that long-term (16 weeks) treatment with amlodipine is the most effective in antagonizing BP-linked oxidative stress in the treatment groups by at least partially increasing SOD and repressing gp91^phox^.

### Changes in the levels of angiotensin II (Ang II) and aldosterone (Ald) in the cerebral cortex of the six groups

**Figure 5:**
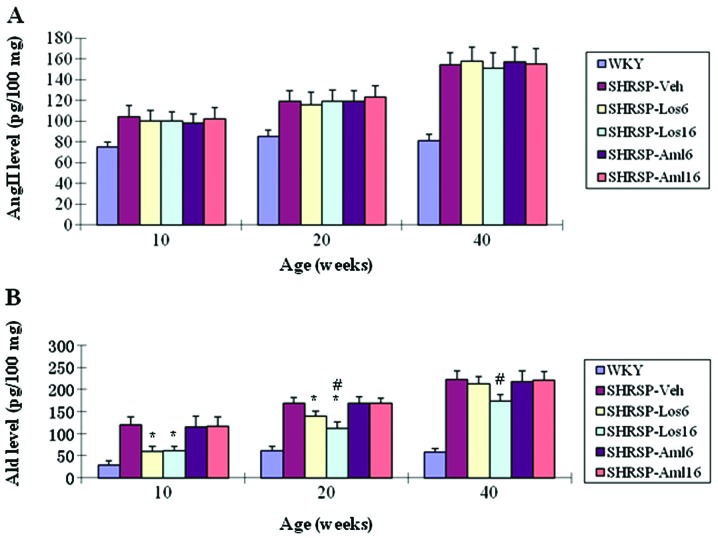
Changes in the levels of angiotensin II (Ang II) and aldosterone (Ald) in the cerebral cortex of Wistar Kyoto rats (WKY), stroke-prone spontaneously hypertensive rats (SHRSP)-Veh and the four drug treatment groups at different ages (weeks). (A) Ang II was measured using radioimmunoassay on brain tissues collected from each of these groups. No significant changes were observed between SHRSP-Veh and any of the four drug treatment groups. n=8/group. (B) Ald was measured using radioimmunoassay on brain tissues collected from each of these groups. ^*^P<0.05, compared with SHRSP-Veh; ^#^P<0.05, compared with SHRSP-Los6. The comparisons were made between groups of the same age as indicated. Note that there is a significant difference between WKY and any of the SHRSP groups.

The local activity of renin-angiotensin system (RAS) was also involved in BP-associated target organ damage (20,21). To examine whether these prehypertension treatments altered RAS activity in brain, we first measured the levels of Ang II and Ald in the brains obtained from WKY and SHRSP of each group. As shown in Fig. 5A, each of these treatments did not lower Ang II levels in the SHRSP brains compared with SHRSP-Veh, however, Ald levels were significantly downregulated in SHRSP-Los6 and SHRSP-Los16 at 10 and 20 weeks compared with SHRSP-Veh, and remained low in SHRSP-Los16 up to 40 weeks (Fig. 5B). However, short- or long-term treatment with amlodipine did not effectively affect the brain Ald compared with SHRSP-Veh (Fig. 5B). Taken together, losartan is a preferred alternative compared with amlodipine in decreasing brain Ald levels. Additionally, long-term treatment with losartan is required to maintain local Ald at low levels for the long-term.

### Changes in the expression levels of AT1R and AT2R in the cerebral cortex of the six groups

**Figure 6:**
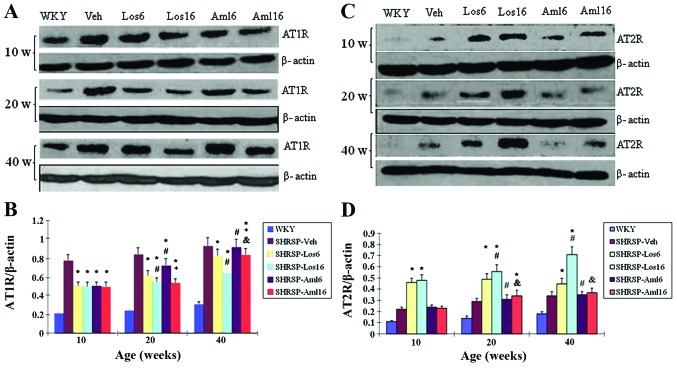
Changes in the expression levels of AT1R and AT2R in the cerebral cortex of Wistar Kyoto rats (WKY), stroke-prone spontaneously hypertensive rats (SHRSP)-Veh and the four drug treatment groups at different ages (weeks). (A and B) Western blot was performed on protein lysates extracted from brains of rats from each group at different ages as indicated (A). β-actin served as the control. (B) Statistical analysis of the results of (A). n=8/group. (C and D) Western blot analysis was performed as in (A), but the blot was reacted with anti-AT2R antibody (C). (D) Statistical analysis of the results of (C). n=8/group. ^*^P<0.05, compared with SHRSP-Veh; ^#^P<0.05, compared with SHRSP-Los6; ^&^P<0.05, compared with SHRSP-Los16; ^+^P<0.05, compared with SHRSP-Aml6. The comparisons were made between groups of the same age as indicated. Note that there is a significant difference between WKY and any of the SHRSP groups.

Hypertension has been associated with changes in the AT2R/AT1R ratio (22,23). To determine whether the short- and long-term administration of losartan and amlodipine for treatment of prehypertension also caused changes in the AT2R/AT1R ratio in brain, western blots were performed on protein lysates purified from the brains of WKY, SHRSP-Veh, and the four treatment groups at 10, 20, and 40 weeks, respectively. As shown in Fig. 6A, at 10 weeks, all four treatment groups showed a significant decrease in AT1R compared with SHRSP-Veh (P<0.05), however, no significant difference among the treatment groups was observed. At 20 weeks, the decrease in the AT1R levels in all four treatment groups was maintained (P<0.05, vs. SHRSP-Veh). However, a more significant decrease was only observed in the two long-term treatment groups as compared to the two short-term treatment groups (P<0.05, SHRSP-Los16 vs. SHRSP-Los6; P<0.05, SHRSP-Aml16 vs. and SHRSP-Aml6), although SHRSP-Los6 was more effective in reducing AT1R levels than SHRSP-Aml6 (^#^P<0.05). At 40 weeks, persistent lower AT1R levels were observed in all the treatment groups with the exception of SHRSP-Aml6, with SHRSP-Los16 exhibiting the best effects (P<0.05, SHRSP-Los16 vs. SHRSP-Los6; P<0.05, SHRSP-Los16 vs. SHRSP-Aml16).

For AT2R, at 10 weeks, the losartan treatment groups showed a significant increase compared with SRHSP-Veh (P<0.05), unlike the amlopidine treatment groups. At 20 weeks, the two losartan treatment groups continued to show a substantial increase in AT2R, and SHRSP-Aml16 showed a slight but significant increase in AT2R in brain compared with SHRSP-Veh (P<0.05). Compared with SHRSP-Aml16, 16 weeks of treatment with losartan showed elevated levels of AT2R in brain (P<0.05). During this period, a time-dependent increase in AT2R by losartan treatments was also observed (P<0.05, SHRSP-Los16 vs. SHRSP-Los6). For the same length of time of treatment, losartan showed higher impacts on AT2R than amlodipine (P<0.05). At 40 weeks, the pattern of the difference in AT2R levels in these groups was identical to that observed at 20 weeks, except that the levels of AT2R of SHRSP-Aml16 returned to the comparable level of that of the SHRSP-Veh group. Taken together, these data demonstrated that long-term (16 weeks) treatment with losartan has the most beneficial effects on suppressing AT1R levels and elevating AT2R levels.

## Discussion

In recent years much attention has been paid to prehypertension as it can cause multiple target organ damage such as hypertension. In a recent study, it was suggested that prehypertension was linked to brain structural damage in humans (7). To investigate whether controlling prehypertension by short- or long-term drug administration would benefit brain structure as well as prevent stroke, and to explore which drug, losartan (AT1R blocker) or amlodipine (calcium channel blocker), had more protective effects on brain when used for prehypertension treatment, we utilized the well-established SHRSP as a model system. As with SHR, SHRSP has a relatively low BP at the age of 4 weeks. The BP progressively increases between 4 and 10 weeks, and peaks between 10 and 12 weeks. Thus, BP of SHRSP at ages of 4–10 weeks is comparable to clinically defined prehypertension. Those studies suggest that losartan is a preferred candidate drug as comapred to amlodipine to prevent brain from prehypertension-associated damage for long-term treatment in the clinic.

A complete RAS is present in brain (24,25), which plays important roles in maintaining normal function and the pathogenesis of brain (20,21). Short-term treatment with the AT1R blocker candesartan on SHRSP during the prehypertension period prevented hypertensive end organ damage, including cerebral edema, after maturation, by at least partially inhibiting local RAS (26,27). Consistent with those reports, the present study has demonstrated that short- and long-term treatments with losartan effectively reduced brain damage such as edema and bleeding, and greatly improved clinical scores of stroke compared with amlodipine treatment groups. Although short- and long-term losartan treatments did not significantly affect Ang II levels in the cerebral cortex, they substantially reduced Ald levels in the brain, with the long-term treatment achieving reduced levels compared with the short-term treatment after maturation. Additionally, losartan treatments greatly decreased AT1R and increased AT2R in the brains of SHRSP compared with amlodipine treatments. AT1R and AT2R mediates the biological functions of Ang II, and it is generally believed that AT2R functionally antagonized AT1R (28). Moreover, a decrease in AT1R and an increase in AT2R was reported to reduce hypertensive end organ damage (29,30). In addition, the specific activation of AT2R by its agonist protected high BP-associated organ damage without exerting significant antihypertensive effects (30). Obviously, the long-term (16 weeks) losartan treatment had more sustained impacts on AT1R/AT2R ratio than the short-term (6 weeks) treatment, and exerted the most beneficial effects on brain structure as evidenced by alleviated edema and bleeding compared with the other three treatment groups. Given the above findings, it is highly likely that one of the mechanisms underlying beneficial effects on brain resulting from long-term losartan treatment is to suppress AT1R-mediated local RAS activity and increase AT2R levels in cerebral cortex.

Compared with the effective roles losartan played in inhibiting local RAS activity, the short- and long-term treatments with amlodipine were not as effective as losartan. Particularly, the levels of AT2R in the brains of SHRSP following long-term (16 weeks) treatment with amlodipine increased at 20 weeks but returned to the levels equivalent to that of SHRSP-Veh at 40 weeks. Thus, amlodipine, unlike losartan, is not capable of persistently repressing the local RAS activity, which is important for the protection of BP-related end-stage brain damage. Consequently, losartan is a better drug than amlodipine for treatment of prehypertension in order to prevent mature stage cerebrovascular damage.

In contrast to the impotent roles amlodipine played in reducing local RAS activity, amlodipine decreased brain cell apoptosis of SHRSP more effectively at the ages of 10 and 20 weeks compared with losartan, although this effect was poorly sustained at the age of 40 weeks. Increased apoptosis was observed in a number of age-related diseases (31), including neurodegenerative disorders (32). More recent studies indicated that prehypertension was associated with premature aging (7), although whether this was attributable to enhanced apoptosis is not clear. One-year-old hypertensive mice also exhibited elevated apoptosis in brain (33). Thus, elevated apoptosis is assumed to be a common phenomenon in BP-associated brain pathophysiology. Mechanistically, oxidative stress was one of the factors that promoted apoptosis (34). In the present study, we measured the local levels of the factors gp91^phox^ and SOD, which are involved in regulating oxidative stress. At 10 and 20 weeks, losartan and amlodipine significantly decreased gp91^phox^ and increased SOD in the brains of SHRSP while substantially suppressing apoptosis, suggesting a potential connection of decreased oxidative stress to decreased apoptosis by treatment. Notably, at 40 weeks, the long-term treatments with losartan and amlodipine persistently inhibited gp91^phox^ and elevated SOD, while they failed to repress apoptosis as was the case at 10 and 20 weeks. One possible explanation for this phenomenon is that at early stages, increased apoptosis in the brain of SHRSP is closely associated with increased oxidative stress, but at later stages such as 40 weeks, increased oxidative stress contributes little to hypertension-associated apoptosis in brain. The exact mechanisms involved in hypertension-linked apoptosis in brain at early and late stages should be investigated. Considering the findings that i) amlodipine treatment inhibited apoptosis more effectively than losartan but was less effective in preventing brain damage, ii) at 40 weeks there was no significant difference in apoptosis between SHRSP-Los16 and SHRSP-Veh, and iii) 16 weeks losartan treatment most effectively prevented SHRSP from stroke and improved brain function as evidenced by the greatly improved clinical scores of stroke, we conclude that suppressing apoptosis is not a significant mechanism by which losartan substantially improved brain function.

In summary, we evaluated whether prehypertensive treatment with different drugs (losartan and amlodipine) and different lengths of treatment time (6 and 16 weeks) affected the function and pathology of SHRSP brains up to 40 weeks, and the associated mechanisms. Amlodipine is more effective in inhibiting apoptosis and oxidative stress, however, losartan is more potent in decreasing BP, repressing local RAS activity and increasing local AT2R levels, leading to more protection from hypertension-associated brain damage such as stroke. Findings of this study provide evidence for the clinical selection of optimal drugs for prehypertension treatment to protect BP-associated end stage cerebrovascular diseases.

## Figures and Tables

**Table I:** Summary of clinical scores of stroke of WKY, SHRSP-Veh and four drug treatment groups.

Evaluation level	WKY n=8	SHRSP-Veh n=5	SHRSP-Los6 n=8	SHRSP-Los16 n=8	SHRSP-Aml6 n=6	SHRSP-Aml16 n=7
0	8	0	3	6	0	0
1	0	0	5	2	0	0
2	0	0	0	0	1	2
3	0	2	0	0	3	4
4	0	3	0	0	2	1
Mean ranking	9	36.5^a^	16.5^a^,^b^	12^a^,^b^	33.5^a^,^c^	31.36^a^,^c^

The Kruskal-Wallis H test was used to analyze the significance of data distribution in the six groups, and the Mann-Whitney U test was used for comparison between two groups as indicated.

a P<0.05, compared with WKY;

b P<0.05, compared with SHRSP-Veh;

c P>0.05, compared with SHRSP-Veh.

WKY, Wistar Kyoto rats; SHRSP, stroke-prone spontaneously hypertensive rats.
